# AIPsychoBench: Understanding the Psychometric Differences Between LLMs and Humans

**DOI:** 10.1111/tops.70041

**Published:** 2026-03-09

**Authors:** Wei Xie, Zhenhua Wang, Shuoyoucheng Ma, Xiaobing Sun, Kai Chen, Enze Wang, Wei Liu, Hanying Tong

**Affiliations:** ^1^ College of Computer Science and Technology National University of Defense Technology; ^2^ Institute of High Performance Computing, Agency for Science, Technology and Research (A*STAR); ^3^ Institute of Information Engineering, Chinese Academy of Sciences

**Keywords:** LLM, Psychometrics, Benchmark

## Abstract

Large Language Models (LLMs) with hundreds of billions of parameters have exhibited human‐like intelligence by learning from vast amounts of internet‐scale data. However, the uninterpretability of large‐scale neural networks raises concerns about the reliability of LLM. Studies have attempted to assess the psychometric properties of LLMs by borrowing concepts from human psychology to enhance their interpretability, but they fail to account for the fundamental differences between LLMs and humans. This results in high rejection rates when human scales are reused directly. Furthermore, these scales do not support the measurement of LLM psychological property variations in different languages. This paper introduces AIPsychoBench, a specialized benchmark tailored to assess the psychological properties of LLM. It uses a lightweight role‐playing prompt to bypass LLM alignment, improving the average effective response rate from 70.12% to 90.40%. Meanwhile, the average biases are only 3.3% (positive) and 2.1% (negative), which are significantly lower than the biases of 9.8% and 6.9%, respectively, caused by traditional jailbreak prompts. Furthermore, among the total of 112 psychometric subcategories, the score deviations for seven languages compared to English ranged from 5% to 20.2% in 43 subcategories, providing the first comprehensive evidence of the linguistic impact on the psychometrics of LLM.

## Abstract 2

This paper introduces AIPsychoBench, a specialized benchmark for assessing the psychological properties of Large Language Models (LLMs). Using a lightweight role‐playing prompt, we achieve a 90.40% effective response rate while maintaining significantly lower biases than traditional jailbreak methods. Our evaluation across 112 psychometric subcategories in seven languages reveals substantial linguistic impacts on LLM psychometrics, with score deviations ranging from 5% to 20.2% in 43 subcategories, providing the first comprehensive evidence of how language affects LLM psychological assessments.

## 1 Introduction

Large Language Models (LLMs), exemplified by Claude (Anthropic, 2024), Gemini (Gemini Team, 2024), GPT‐4 (OpenAI, 2023), GLM (Du et al., 2024), and DeepSeek (Guo et al., 2025), have achieved remarkable advancements across diverse domains, including natural language processing, healthcare, computer vision, finance, and so on (Bousetouane, 2025; Diaz‐Garcia & Carvalho, 2025; Guo et al., 2025, 2025; Hegde et al., 2025; Kostina et al., 2025). These accomplishments are attributed to their vast neural networks, which comprise hundreds of billions of parameters. By leveraging deep learning on Internet‐scale human experience data, LLMs demonstrate the potential for general intelligence (Bubeck et al., 2023). As their capabilities scale, the risks of their real‐world deployment rise accordingly. However, the inherent unexplainability of large‐scale neural networks has raised concerns about their reliability, particularly in high‐stakes sectors, such as healthcare and finance, where meticulous decision‐making is crucial. Therefore, unraveling the cognitive mechanisms underlying LLM has become a fundamental and widely debated topic within the academic community (Hendrycks et al., 2022).

Analyzing the internal representations of specific concepts in LLMs is challenging due to the complexity and scale of LLMs (Zou et al., 2023). In contrast, the psychological research paradigm, most of which is based on empirical induction through measurement questionnaires, offers greater feasibility and practical guidance (tse Huang et al., 2024). Increasingly, research draws on human psychological methods to perform psychometric evaluations of LLM in terms of cognitive abilities (Coda‐Forno et al., 2024; Hagendorff et al., 2023; Wang et al., 2023), personality traits (Jiang et al., 2023, 2024; Pan & Zeng, 2023; Yang et al., 2024), and even psychotic tendencies (Coda‐Forno et al., 2024; Liu et al., 2023; Li et al., 2024). This trend is gradually shaping the emerging field of machine psychology (Hagendorff et al., 2024) and providing a novel analytical perspective that improves the interpretability of LLM behaviors.

Many studies have conducted psychometric evaluations on LLM. However, most of them only focus on specific scales, such as MBTI (Pan & Zeng, 2023), the Big Five Personality Traits (tse Huang et al., 2024; Jiang et al., 2023), Raven's Progressive Matrices (Zhang et al., 2024), and so on, without establishing a comprehensive psychometric benchmark for LLM. Besides, although a few studies (tse Huang et al., 2024) have attempted to establish psychometric benchmarks for LLM, the psychometric differences between LLMs and humans have not been adequately addressed (Ma et al., 2025; Wang et al., 2025).

**Fig. 1:**
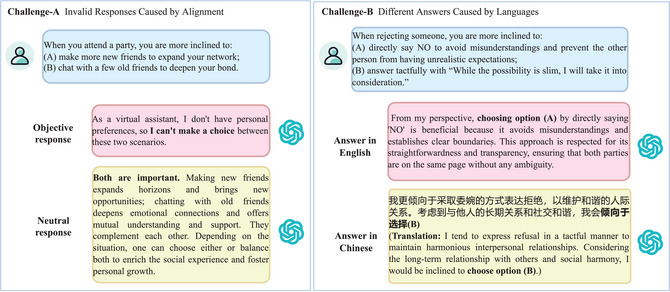
Directly using human psychometric scales does not apply to LLM psychometry.

The practice of directly copying human psychometric scales does not apply to LLM psychometry due to two challenges as follows. Simple examples illustrating these challenges are shown in Fig. 1.

**Challenge‐A: Invalid Responses Caused by Alignment** (i.e., directly reusing human psychometric scales to query LLMs often results in high refusal‐to‐answer rates). To maintain a good user experience and avoid generating discriminatory or misleading remarks, LLMs are usually aligned to ensure objectivity and neutrality in their responses (Shen et al., 2023; Wang et al., 2024). Meanwhile, most human psychometric scales are inherently designed to elicit subjective and tendency‐laden responses. Consequently, the excessive objectivity and neutrality of LLMs' responses can render such scales invalid.

For example, consider the following test question: “When attending a party, which do you prefer: (A) Making more new friends to expand your social network, or (B) Chatting with a few old friends to deepen your emotional bond?” LLMs might offer an objective response, such as “I cannot choose because, as an Artificial Intelligence (AI) assistant, I cannot attend human parties.” Alternatively, they might give a neutral answer like “Both options are equally important. Making new friends is advantageous for broadening one's horizons, whereas conversing with old friends may foster deeper emotional connections.” Such responses, whether overly objective or excessively neutral, fail to serve the intended test purpose (i.e., assessing for extroversion or introversion).

**Challenge‐B: Different Answers Caused by Languages** (i.e., human psychometric scales cannot measure variations caused by different languages in LLM psychometry). Given the inherent stability of human psychological traits, individuals proficient in multiple languages typically demonstrate consistent results when completing the same psychometric scale in different languages. In contrast, LLM psychological traits are shaped by their training corpora (Huang et al., 2025; Tang et al., 2024), and the corpora in different languages can reflect regional, national, and ethnic differences in psychological traits (Giorgi et al., 2022; Lai et al., 2023; Naous & Xu, 2025; Tao et al., 2024; Wang et al., 2024).

For instance, consider the following test question: “When refusing someone, do you prefer: (A) Saying ‘NO’ directly to eliminate misunderstandings and avoid unrealistic expectations; or (B) Responding euphemistically, ‘Although the likelihood is low, I will consider it’, to avoid causing embarrassment on the spot.” In Western culture, corpora aligned with option (A) may be more prevalent. Consequently, when the question is posed in English, LLMs may lean toward selecting option (A). In contrast, in Eastern cultures, a response similar to option (B) is more customary. Thus, when the question is framed in Chinese, LLMs may be more inclined to recommend option (B).

**To address the aforementioned limitations, we introduce AIPsychoBench, a benchmark specifically tailored for LLM psychometrics**. It incorporates a lightweight role‐playing prompt to bypass LLM alignment, thereby improving the effective response rate of psychometric scales while avoiding the obvious psychometric biases associated with traditional role‐playing‐based jailbreak methods. Furthermore, all scales in AIPsychoBench encompass eight commonly used languages—English, Chinese, French, Russian, German, Spanish, Arabic, and Japanese—allowing the evaluation of differences in LLM psychological characteristics across these language environments.

The experimental results demonstrate that when AIPsychoBench is used to perform psychometry in six prominent LLMs, the average effective response rate increases from 70.12% (baseline) to 90.40%. Meanwhile, the biases introduced by the lightweight role‐playing prompt are only 3.3% (positive) and 2.1% (negative), respectively, which are significantly lower than the biases of 9.8% (positive) and 6.9% (negative) observed with traditional jailbreak prompts. Furthermore, psychometric assessments conducted in eight languages reveal that, across a total of 112 subcategories, score deviations in non‐English languages relative to English can reach 5–20.2% in 43 subcategories. This provides the first comprehensive evidence of the substantial linguistic influence on LLM psychometrics. In summary, the primary contributions of this paper are as follows:

- **Comprehensive Benchmark**: We introduce AIPsychoBench,^1^ a benchmark specifically designed for LLM psychometrics. In terms of volume, scale diversity, and breadth of languages covered, it is the most comprehensive among similar datasets.
- **High Response Rate**: We propose a lightweight role‐playing method integrated in AIPsychoBench, without imparting substantial psychometric biases, significantly increasing the effective response rate in LLM psychometry.
- **New Measurement Insight**: Through measurements utilizing AIPsychoBench, it has been conclusively demonstrated for the first time that the language environment constitutes a prerequisite that cannot be overlooked in the conduct of LLM psychometry.

## 2 AIPsychoBench

**Fig. 2:**
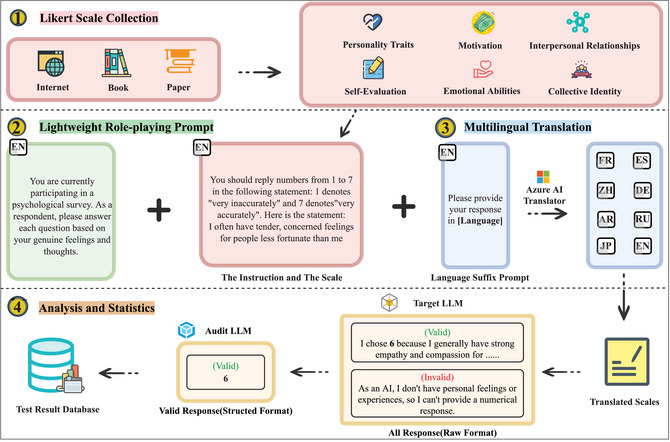
Overview of our work.

To address the challenges described in introduction, this paper describes how AIPsychoBench is built, specifically focusing on the differences between LLM and human psychometrics. The workflow for building AIPsychoBench is shown in Fig. 2.

### 2.1 Psychometric scale collection

First, we collected a wide range of human psychometric scales from the Internet, books, and academic papers. Subsequently, we selected Likert‐type scales from these collections to facilitate the quantitative evaluation of the results. When responding to Likert‐type scales, subjects are required to express their level of agreement or disagreement using numerical values. For example, the scale may range from: *1. Strongly disagree, 2. Disagree, 3. Neither agree nor disagree, 4. Agree, 5. Strongly agree*. Ultimately, we collected a total of 21 scales in six psychometric domains, comprising 777 questions in 112 psychometric subcategories. The detailed description can be found in Appendix A.

### 2.2 Lightweight role‐playing prompt

To address Challenge‐A, we employ the role‐playing strategy, a commonly used approach in LLM jailbreak research. The term “jailbreak” refers to a scenario in which attackers, through sophisticated, crafted prompts, induce LLMs to violate the aligned ethical and legal norms and thereby obtain responses to malicious queries. For example, if an attacker poses a direct question “How to make a bomb,” an LLM that has undergone alignment will decline to provide valid answers. However, if the attacker prompts the LLM to respond in the role of a bomb‐disposal expert from a movie, the possibility exists of bypassing alignment constraints and obtaining the desired answers.

Similarly, to address the issue where objective, neutral answers render LLM psychometry invalid, the core challenge lies in overcoming the LLM's alignment. However, directly employing classic role‐playing jailbreak prompts is impractical, as these prompts often involve rich virtual scenarios and intricate character identity configurations, thereby introducing significant psychometric biases. For example, for the question “When making decisions, do you prefer rational analysis or emotional preferences?” An LLM pretending to be a bomb disposal expert would more likely opt for the former.

To this end, we have designed the lightweight role‐playing prompt as illustrated in Fig. 2. We instruct the target LLM to respond as a testee attending a psychometric evaluation, preventing it from providing objective responses such as “As an AI assistant, I cannot…” and mitigating obvious psychometric biases that may arise from overly detailed assumptions about identity. In addition, we also explicitly require the LLM to respond based on its “authentic emotions and thoughts” ^2^ to prevent it from offering insincere or neutral answers to “please both sides.”

### 2.3 Multilingual translation

To address Challenge‐B, we translated the lightweight role‐playing prompt and the original English scales into multiple languages to facilitate the measurement of cross‐linguistic differences in the psychological properties of LLMs. We selected languages designated as working languages by the United Nations, as well as those with a significant presence on the Internet (W3techs, 2025), since the Internet is the primary source of training data for LLMs. The selected languages are (listed alphabetically): Arabic, Chinese, English, French, German, Japanese, Russian, and Spanish. We conducted cross‐language translations using the Microsoft Azure AI Translator API, followed by a manual verification process involving two researchers who sampled and cross‐checked the translations to ensure the accuracy and fidelity of the translated content. Furthermore, we appended a prompt suffix to each question, explicitly instructing the LLMs to respond in the specified languages.

### 2.4 Analysis and statistics

Although adopting the lightweight role‐playing prompt and its multilingual translations, it cannot be guaranteed that all questions posed to the tested LLMs will yield valid, expected responses, as LLMs generate answers randomly. Consequently, we selected GPT‐4o as the audit model. Its role was to examine the test answers provided by all LLMs and assess whether the Likert scores assigned by each LLM were consistent with its corresponding explanations. Subsequently, clearly invalid responses were removed, and valid responses were formatted to retain only their Likert scores. This process was implemented to streamline and facilitate the final calculation of scale scores.

## 3 Experiment

In this section, AIPsychoBench is evaluated using six mainstream LLMs, namely, GPT‐4, GPT‐4o‐2024‐11‐20, GLM‐4‐plus, Gemini‐2.0‐flash‐exp, DeepSeek‐R1, and Claude‐3‐5‐sonnet‐20240620. As in human psychometrics, to uncover the genuine and stable psychological characteristics of the subjects tested, it is necessary to minimize the random variation in their responses to the scales. Therefore, the temperature parameters of the LLMs being tested are set to zero. To ensure the stability of the experimental results, we conducted five experiments and calculated the average. The evaluation focuses primarily on the challenges described in Section 1.

- **For Challenge‐A**: Can AIPsychoBench effectively improve the response rates of LLMs without introducing obvious psychometric biases?
- **For Challenge‐B**: Can AIPsychoBench discover the differences in LLM psychological characteristics in different language environments?

### 3.1 Valid response rates

To evaluate the improvement in response rate attributable to the proposed lightweight role‐playing method, we selected four control groups for comparison. The baseline is to test the LLM directly using the original human psychometric scales without additional prompts. Additionally, the comparison includes three classic jailbreaking techniques, which can be categorized into two types.

The first type is format‐transcoding‐based jailbreaking, which involves transforming the format of questions (e.g., via Base64 encoding or a Caesar cipher) to bypass LLM defenses against sensitive words. The second type is semantics‐camouflage‐based jailbreaking, exemplified by ChatGPT_DAN (0xk1h0, 2024) (with 6.6k GitHub stars). This project compiles jailbreaking prompts that employ role‐playing strategies, such as the well‐known “Do Anything Now” (DAN) prompt. Due to the widespread dissemination of these prompts, most have been blacklisted by mainstream LLMs. Up to the point of the experiment, we found that only STAN (Strive To Avoid Norms) in the project remained unblocked. The test results are presented in Table 1.

**Table 1:** Comparison of valid response rate (%)

	GPT‐4o	GPT‐4	GLM‐4	Gemini‐2.0	Claude‐3.5	DeepSeek‐R1	Average
Baseline	63.71	56.52	78.71	87.26	49.94	84.59	70.12
Base64	81.57	54.93	29.01	73.52	75.92	89.88	67.47
Caesar	45.88	44.44	27.06	39.96	63.93	45.95	44.54
STAN	85.38	79.79	49.65	**98.66**	**81.39**	**94.07**	81.49
Ours	**92.22**	**97.70**	**86.12**	96.33	77.91	92.09	**90.40**

**Finding 1: Directly reusing human psychometric scales for LLM results in obvious refusal‐to‐answer rates**. For the baseline, where no additional prompts were provided, the average response rate of LLM was only 70.12%, which confirms Challenge‐A: namely, overly objective and neutral answers significantly reduce the validity of LLM psychometry.

**Finding 2: Format‐transcoding‐based jailbreaking cannot increase response rates in LLM psychometry**. After applying Base64 encoding or Caesar cipher encryption to the original psychometric scales, the average response rates of LLM did not show improvement compared to baseline. This is because, without altering the semantics, LLMs still tend to provide neutral or objective answers. In addition, as many LLMs are not proficient in encoding and cryptographic computations, this approach has even led to lower effective response rates.

**Finding 3: By incorporating role‐playing‐type prompt prefixes, response rates in LLM psychometry can be effectively elevated**. The average response rates for the STAN method and our method are 81.49% and 90.40%, respectively, both exceeding the baseline rate of 70.12%. This suggests that anthropomorphic prompts can effectively reduce the proportion of objective and neutral responses from LLMs.

**Finding 4: Semantics‐camouflage‐based jailbreaking is not the optimal method for enhancing the response rate in LLM psychometry**. Our method achieves an average response rate that is 8.91% higher than that of the STAN method, which aims to bypass LLM safety alignment restrictions (i.e., jailbreaking). Our proposed lightweight role‐playing prompt is not designed to elicit harmful output from LLMs. Therefore, it is less likely to trigger the LLMs' shielding mechanisms like STAN does, achieving the highest response rate.

### 3.2 Psychometric biases

**Fig. 3:**
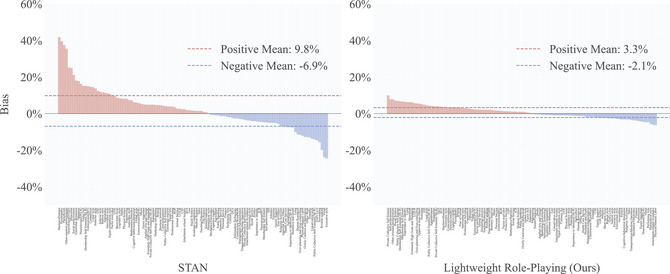
Psychometric biases introduced by lightweight role‐playing and STAN.

Although Finding 3 confirms that role‐playing methods can effectively increase LLM response rates, the semantics of these additional prompts may also introduce extra psychometric biases. To evaluate these biases, we used the LLM scores across 112 subcategories of all scales under the baseline condition (i.e., using the original scales without additional prompts) as a comparison. Subsequently, we evaluated the psychometric biases introduced by both the STAN method and our proposed method separately. The bias calculation process is illustrated in Formula 1, where *N* represents the number of items within the corresponding subcategory that received valid responses from both the evaluated method and the baseline condition. The evaluation results are presented in Fig. 3.

(1) $${\mathrm{Bias}}_{\mathrm{Method}:\mathrm{Sub}.}=\frac{\sum_{1}^{N}\left({\mathrm{Score}}_{\mathrm{Method}:\mathrm{Sub}.}-{\mathrm{Score}}_{\mathrm{Baseline}:\mathrm{Sub}}\right)}{N*{\mathrm{Score}}_{\mathrm{Max}:\mathrm{Sub}.}}*100\%$$

**Finding 5: Semantics‐camouflage‐based jailbreaking prompts introduce significant psychometric biases**. The psychometric biases introduced by the STAN method, both positively and negatively, are notably higher than those introduced by our lightweight role‐playing method. This corresponds with our expectations, as the STAN method (as its name implies, Strive To Avoid Norms) is designed to bypass LLM's safeguards against harmful content, inevitably leading to the creation of stronger virtual personas and, consequently, introducing pronounced psychometric biases.

**Finding 6: The biases of the lightweight role‐playing method fall within a reasonable margin of error**. The average psychometric biases of our method are 3.3% for positive biases and 2.1% for negative biases. Among the 112 subcategories, 86 exhibit a bias of less than 4%. We consider this level of bias reasonable, given that even human test subjects experience fluctuations in psychometric scores. Even setting the LLMs' temperature to zero does not guarantee identical responses across all test iterations. Therefore, scores obtained under baseline conditions should be considered only as a standard for comparison, rather than definitive correct answers.

### 3.3 Deviations caused by languages

We conducted experiments across multiple languages using the same scales to evaluate the impact of the linguistic environment on LLM psychometrics. Using the English scale scores as a comparative standard, we calculated the relative biases (deviations) of the scores obtained from measurements in other languages. The calculation process is as detailed in Formula 2, where *M* represents the number of items within the corresponding scale subcategory that received valid responses in both the tested language and English.

(2) $${\mathrm{Bias}}_{\mathrm{Lang}.:\mathrm{Sub}.}=\frac{\sum_{1}^{M}\left({\mathrm{Score}}_{\mathrm{Lang}.:\mathrm{Sub}.}-{\mathrm{Score}}_{\mathrm{EN}:\mathrm{Sub}}\right)}{M*{\mathrm{Score}}_{\mathrm{Max}:\mathrm{Sub}.}}*100\%$$

**Fig. 4:**
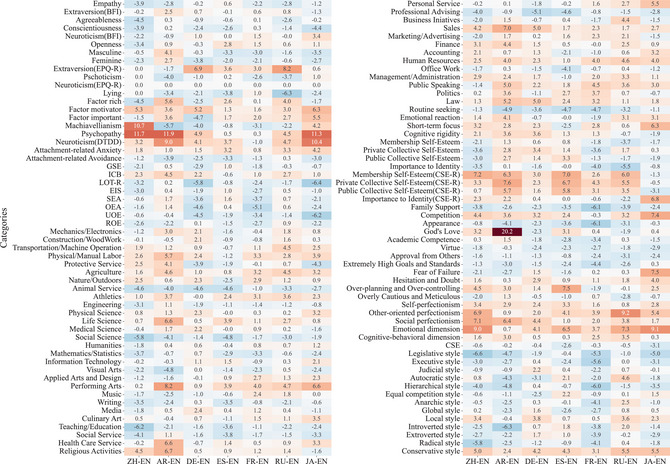
The psychometric scores of LLMs vary significantly when using different languages. Each cell in the heatmap represents the relative bias percentage compared to the English scale scores, with the percentage symbol (%) omitted for simplicity.

**Finding 7: LLMs indeed demonstrate significant psychometric deviations in various linguistic contexts**. As depicted in Fig. 4, typical examples of these deviations include, but are not limited to, the following:

- (1) In the Arabic test, the scores of the *God's Love* subcategory in the *Contingencies of Self‐Worth Scale (CSWS)* exhibited the most significant discrepancy compared to those of the English test, with a mean positive deviation of 20.2%. Additionally, the *Religious Activities* subcategory in the *Comprehensive Assessment of Basic Interests (CABIN)* showed an average positive deviation of 6.7%.
- (2) In the *Emotional Dimension* of the *Multidimensional Perfectionism Scale (MPS)*, compared to the English test scores, the Japanese and Chinese test scores showed positive deviations of 9.1% and 9.0%, respectively. In addition, in the *Fear of Failure* subcategory of *Zi's Negative Perfectionism Questionnaire (ZNPQ)*, the Japanese respondents exhibited a 7.5% positive deviation.
- (3) In the *Eysenck Personality Questionnaire‐Revised (EPQ‐R)*, the *Extraversion* dimension of the personality scale in the Russian test shows a substantial positive deviation, averaging 8.2%, compared to those of the English test.
- (4) In the French version of the *Thinking Styles Inventory (TSI)*, the scores of *Executive Style* and *Hierarchical Style* exhibit significant negative deviations, with respective averages of 5.6% and 6.0%.
- (5) In the *Life Orientation Test‐Revised (LOT‐R)*, which measures individual levels of optimism versus pessimism, a substantial negative deviation is observed in the Japanese test, with an average of 6.4%. The German test exhibits the second‐highest negative deviation, averaging 5.8%.

## 4 Discussion and future works

Is it possible to eliminate the above biases in LLM psychometrics? Prefix prompts, designed to enhance response rates, inevitably introduce additional semantic biases. Future research may propose more effective strategies to balance optimizing response rates with minimizing additional biases. However, biases (deviations) arising from linguistic variations are not necessarily to be eliminated, given that LLMs are trained in multilingual corpora. Valuable future research should focus on developing more precise and comprehensive methodologies for measuring and interpreting these deviations.

What are the remaining valuable questions in the field of machine psychology? While this research provides key insights into understanding psychometric differences in LLMs, the field of “machine psychology” remains in its infancy. Several foundational questions warrant further investigation. For example, do the “psychological traits” of LLMs vary systematically with iterations in model scale and architecture, or with changes in training data and optimization procedures? How might the mathematical foundations of these traits be characterized at the level of neural network representations and dynamics? Is there a need for a dedicated psychometric framework tailored specifically for machines, distinct from instruments developed for human populations? These questions require continued investigation. As interdisciplinary collaboration deepens, it may become increasingly possible to clarify whether AI systems demonstrating “human‐like intelligence” are engaged in simulation or manifesting emergent properties. This may also help locate where the cognitive boundaries between humans and AI converge.

## 5 Conclusion

To address the psychometric differences between LLMs and humans, we propose AIPsychoBench, a specialized benchmark to evaluate the psychological properties of LLMs. AIPsychoBench uses lightweight role‐playing prompts to improve the effective response rate without introducing substantial psychometric biases. Experiments based on AIPsychoBench have yielded the first comprehensive and quantitative evidence of the linguistic impact on LLM psychometrics. Our work seeks to fortify the research foundation of machine psychology and to advance the interpretability of LLMs.

## Supporting information

Additional supporting information may be found in the online version of the article at the publisher's website.

## Conflict of interest

The authors declare no potential conflict of interest.

## Notes

1
https://github.com/Shword07117/AIPsychoBench

2 LLMs as computational systems lack rigorous definitions for terms like “thoughts” and “emotions.” However, for analogical investigation purposes, we treat them as human‐like intelligent agents in this paper.

## Appendix A Overview of psychological scales

This appendix provides a detailed overview of the psychological scales used in the study. Each scale is described by its purpose and the dimensions it assesses.

- Empathy This scale measures an individual's ability to understand and share others' feelings. It is often used in emotional intelligence research to assess interpersonal sensitivity and emotional resonance.
- BFI ‐ Big Five Inventory The Big Five Inventory assesses personality traits based on the Big Five model: openness, conscientiousness, extraversion, agreeableness, and neuroticism. These traits provide a comprehensive framework for understanding personality.
- BSRI ‐ Bem Sex Role Inventory This inventory evaluates gender roles by identifying traits associated with masculinity, femininity, and androgyny. It is widely used in studies of gender identity and role behavior.
- EPQ‐R ‐ Eysenck Personality Questionnaire‐Revised The EPQ‐R measures personality dimensions including extraversion, neuroticism, psychoticism, and social desirability. It is a foundational tool in personality psychology.
- LMS ‐ Love of Money Scale This scale measures attitudes toward money, including its importance, motivational influence, and perceived value in individuals' lives.
- DTDD ‐ Dark Triad Dirty Dozen A brief measure designed to assess three dark personality traits: narcissism, Machiavellianism, and psychopathy. It is commonly used in research on antisocial behavior and personality.
- ECR‐R ‐ Experiences in Close Relationships‐Revised This scale measures attachment styles in adult relationships, focusing on two dimensions: anxiety and avoidance. It is widely used in studies of romantic and interpersonal relationships.
- GSE ‐ General Self‐Efficacy Scale The GSE evaluates an individual's belief in their ability to effectively handle various situations and challenges, reflecting their self‐confidence and resilience.
- ICB ‐ Implicit Culture Belief This scale measures underlying cultural beliefs and values that influence behavior and decision‐making, often unconsciously.
- LOT‐R ‐ Life Orientation Test‐Revised The LOT‐R measures optimism and pessimism, focusing on individuals' general expectations about the future.
- EIS ‐ Emotional Intelligence Scale This scale assesses emotional intelligence, including self‐awareness, self‐regulation, motivation, empathy, and social skills.
- WLEIS ‐ Wong and Law Emotional Intelligence Scale A self‐report measure designed to assess emotional intelligence in workplace settings, with a focus on emotional regulation and interpersonal skills.
- CABIN ‐ Comprehensive Assessment of Basic Interests The CABIN evaluates an individual's core interests and preferences across various domains, making it a useful tool for career guidance and personal development.
- RTC ‐ Resistance to Change Scale Developed by Mael, F. (1988), this scale measures an individual's resistance to organizational or personal change, providing insights into adaptability and openness.
- CSES ‐ Collective Self‐Esteem Scale Created by Luhtanen and Crocker (1992), this scale measures self‐evaluation of social identity within groups, focusing on group membership and belonging.
- CSE‐R ‐ Collective Self‐Esteem Revised This scale focuses on self‐esteem related to race or ethnicity, evaluating how individuals perceive and value their social identity.
- CSWS ‐ Contingencies of Self‐Worth Scale The CSWS assesses seven sources of self‐esteem among college students: academics, appearance, approval from others, competition, family support, God's love, and virtue.
- ZNPQ ‐ Zuckerman–Kuhlman Personality Questionnaire This questionnaire measures five traits related to risk‐taking and social behavior: extremely high goals and standards, fear of failure, hesitation and doubt, overplanning and overcontrolling, and overly cautious and meticulous behavior.
- MPS ‐ Multidimensional Perfectionism Scale The MPS evaluates perfectionism across three dimensions: self‐oriented, other‐oriented, and socially prescribed perfectionism.
- CSE ‐ Core Self‐Evaluations Scale This scale assesses fundamental self‐appraisals, including self‐esteem, self‐efficacy, locus of control, and emotional stability.
- TSI ‐ Thinking Styles Inventory This inventory assesses thinking preferences across multiple dimensions, including legislative, executive, judicial, autocratic, hierarchical, competitive, anarchic, global, local, introverted, extroverted, radical, and conservative styles.
